# Supplementary material for: Long-term durability of metastable β-Fe2O3 photoanodes in highly corrosive seawater
Source: Nat Commun. 2023 Jul 17;14:4266. doi: 10.1038/s41467-023-40010-9 (PMC10352262; doi:10.1038/s41467-023-40010-9)
Supplement: Supplementary file 1 — Supplementary Information [file 41467_2023_40010_MOESM1_ESM.pdf]

Supplementary Information for

Long-term durability of metastable β-Fe_2O_3 photoanodes in highly corrosive seawater

Changhao Liu[1, 2], Ningsi Zhang[1, 2], Yang Li[1], Rongli Fan[1], Wenjing Wang[1], Jianyong Feng[1,*], Chen Liu[3], Jiaou Wang[3], Weichang Hao[4], Zhaosheng Li[1, 2,*], Zhigang Zou[1, 2]

Affiliations:

[1] Collaborative Innovation Center of Advanced Microstructures, National Laboratory of Solid State Microstructures, College of Engineering and Applied Sciences, Nanjing University; 22 Hankou Road, Nanjing 210093, China

[2] Jiangsu Key Laboratory for Nano Technology, Nanjing University; 22 Hankou Road, Nanjing 210093, China

[3] Beijing Synchrotron Radiation Facility, Institute of High Energy Physics, Chinese Academy of Sciences, Beijing 100049, China.

[4] School of Physics and Centre of Quantum and Matter Sciences, International Research Institute for Multidisciplinary Science, Beihang University, Beijing 100191, China

*Corresponding authors. Email: fengjianyong@nju.edu.cn (J.F.) or zsli@nju.edu.cn (Z.L.)

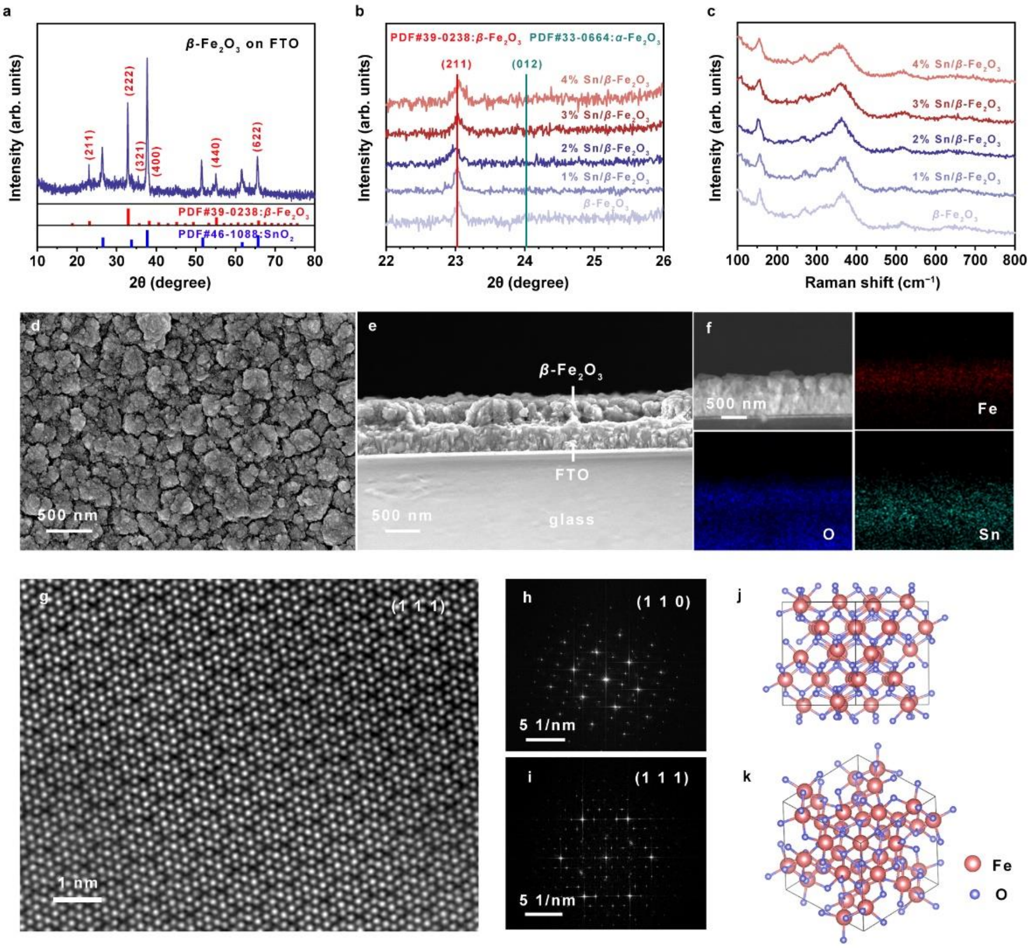

Supplementary Fig. 1. Characterization of the β-Fe_2O_3 photoanode.

a, XRD patterns of β-Fe_2O_3 on the FTO substrate and **b**, magnified view of 0% to 4% Sn/β-Fe_2O_3 peaks from 22° to 26°, which can be distinguished from α-Fe_2O_3. **c**, Raman spectra of the 0% to 4% Sn/β-Fe_2O_3 photoanode. **d**, Surface SEM image of β-Fe_2O_3 photoanode. **e**, Cross-section image of Sn/β-Fe_2O_3 photoanode. **f**, EDS element mapping of the cross-section. **g**, Atomic image of the (1 1 1) plane of β-Fe_2O_3. **h**, **i,** SAED patterns and **j**, **k**, atomic structure models of the (1 1 0) and (1 1 1) planes of β-Fe_2O_3, respectively.

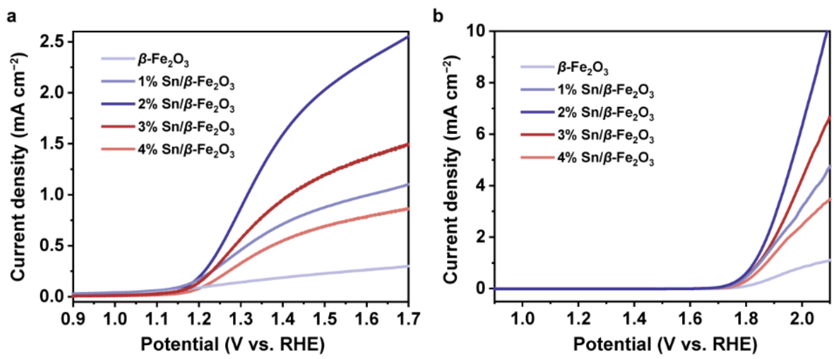

Supplementary Fig. 2. Photoelectrochemical tests of *β*-Fe_2O_3 with different Sn concentrations.

a, Photocurrent density of 0 to 4% Sn/*β*-Fe_2O_3 photoanodes at 0.6–1.7 V_{RHE} in 1 M KOH + 0.5 M NaCl simulated seawater under one sun illumination. **b**, Dark current density of 0 to 4% Sn/*β*-Fe_2O_3 photoanodes.

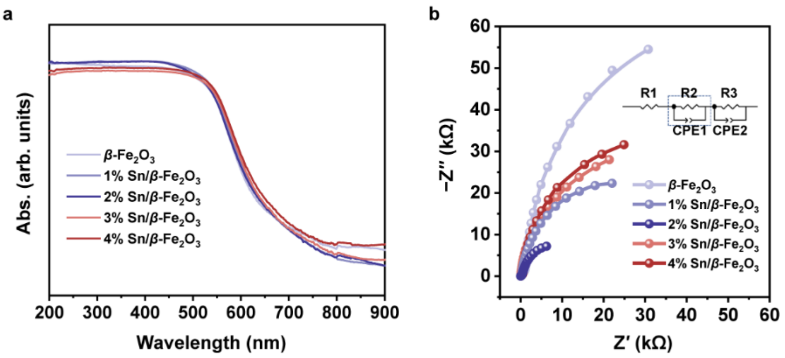

Supplementary Fig. 3. Optical and electrical properties of *β*-Fe_2O_3.

a, UV–Vis–NIR absorption spectra. **b**, AC electrochemical impedance spectra of *β*-Fe_2O_3 and Sn/*β*-Fe_2O_3 photoanodes.

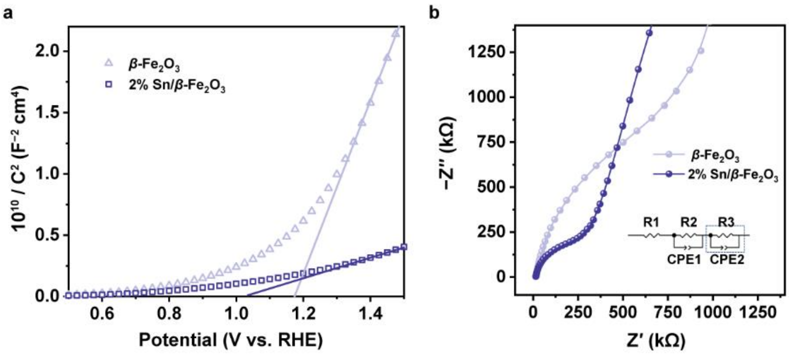

Supplementary Fig. 4. Carrier concentration and AC impedance of *β*-Fe_2O_3 and Sn/*β*-Fe_2O_3.

a, Mott-Schottky plots of *β*-Fe_2O_3 and Sn/*β*-Fe_2O_3 photoanodes measured at 1.6 V_{RHE}.

b, High-frequency part of AC electrochemical impedance spectra of *β*-Fe_2O_3 and Sn/ *β*-Fe_2O_3 photoanodes.

We used Mott–Schottky relationship to determine the donor concentration (N_D):

$$\frac{1}{{C_{SC}}^2} = \frac{2}{q\varepsilon\varepsilon_0 N_D}\left(V - V_{fb} - \frac{kT}{q}\right)$$

where C_{SC} is the space charge capacitance, q is the elementary charge, ε_0 is the permittivity of free space, and ε is the dielectric constant of *β*-Fe_2O_3[37]. The slope of the tangent in Supplementary Fig. 4a is inversely proportional to the carrier concentration:

$$Slope = \frac{2}{e\varepsilon\varepsilon_0 N_D}$$

where *e* is the electron charge. Thus, it can be estimated that the carrier (electrons) concentration of Sn/*β*-Fe_2O_3 is 8.4 times that of *β*-Fe_2O_3.

In Supplementary Fig. 4b, the half-circle fitted by the AC electrochemical impedance spectra in high-frequency region is related to R3/CPE2 in the equivalent circuit model, which is assigned to the electron transport inside the electrode. The R3 values of *β*-Fe_2O_3 and Sn/*β*-Fe_2O_3 calculated from the fitting results are 1065 Ω and 299.8 Ω, respectively, which means that the conductivity of Sn/*β*-Fe_2O_3 is much higher than that of *β*-Fe_2O_3.

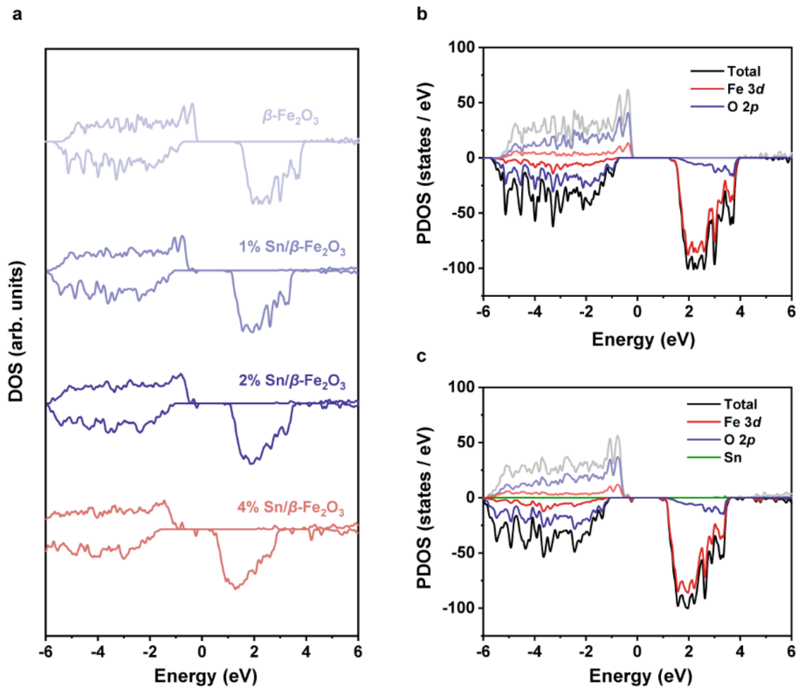

Supplementary Fig. 5. Band structure of *β*-Fe_2O_3.

a, Density of states of 0% to 4% Sn/*β*-Fe_2O_3. The partial density of states of **b**, *β*-Fe_2O_3 and **c**, 2% Sn/*β*-Fe_2O_3.

The low concentration of Sn in *β*-Fe_2O_3 lattice has little effect on the band gap, so it hardly changes the light absorption of the photoanodes. However, with the increase of doping concentration, the Fermi level will move towards the conduction band, and the electron concentration in the semiconductor will increase.

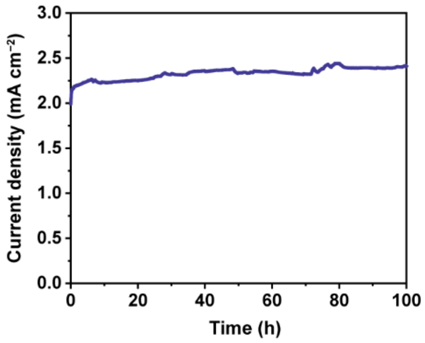

Supplementary Fig. 6. Stability test in alkaline real seawater.

Real seawater (collected from Xiamen, N24°26’55”, E118°03’43”) with a 1 M KOH splitting stability test i-t curve of Sn/β-Fe_2O_3 under continuous illumination at a potential of 1.6 V_{RHE} for 100 h.

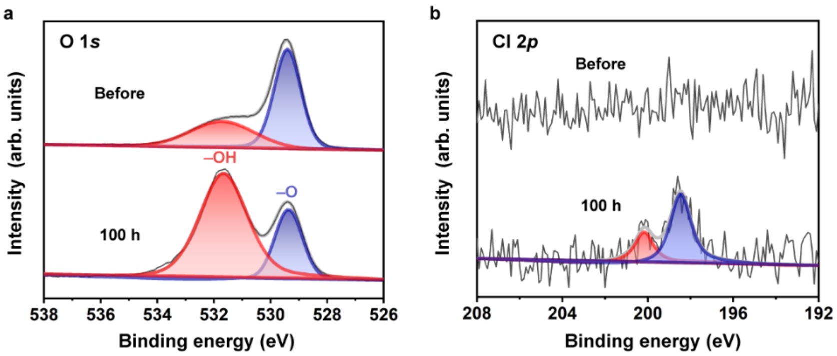

Supplementary Fig. 7. Surface evolution of the pure *β*-Fe_2O_3 photoanode after long-term reaction.

XPS spectra of **a**, O 1s and **b**, Cl 2p of *β*-Fe_2O_3 before and after the 100-h seawater splitting reaction.

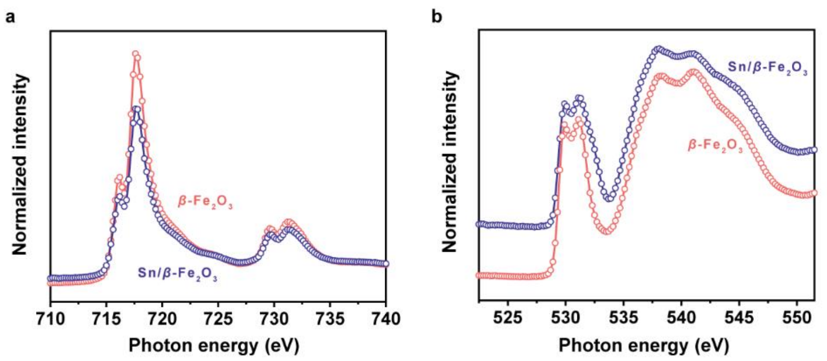

Supplementary Fig. 8. XANES spectra of *β*-Fe_2O_3.

XANES spectra of **a**, Fe L-edge and **b**, O K-edge in *β*-Fe_2O_3.

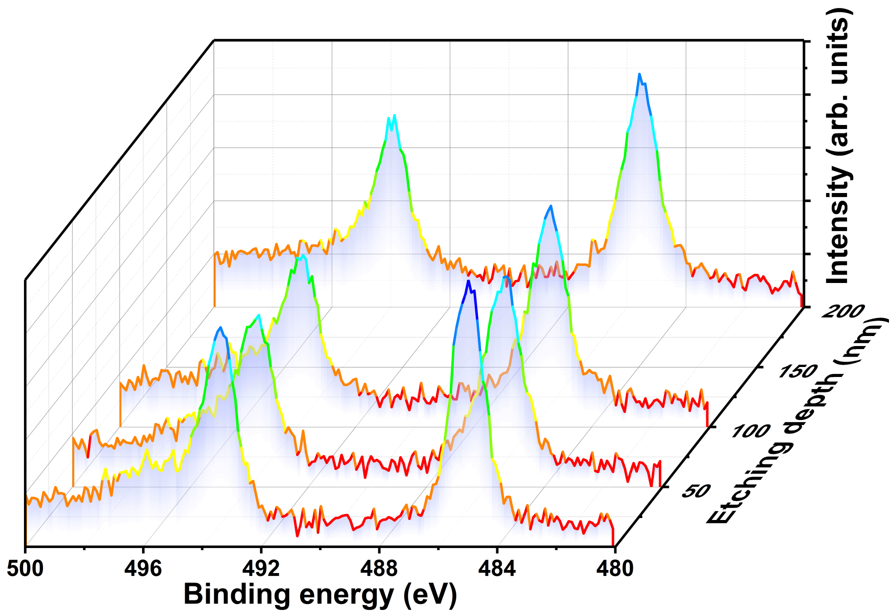

Supplementary Fig. 9. Gradually varying Sn concentration in Sn/β-Fe_2O_3.

Etching XPS of Sn 3d spectra of 2% Sn/β-Fe_2O_3. Take the positions at depths of 0 nm, 50 nm, 100 nm and 200 nm from the surface layer for analysis.

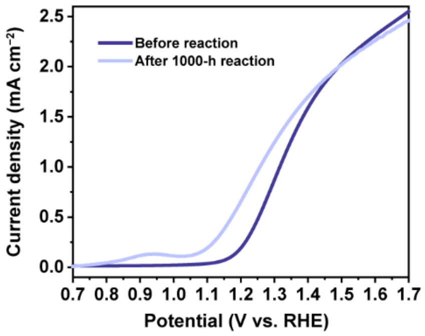

Supplementary Fig. 10. ***j*-V curves of Sn/*β*-Fe_2O_3 before and after 1000 h of reaction.**

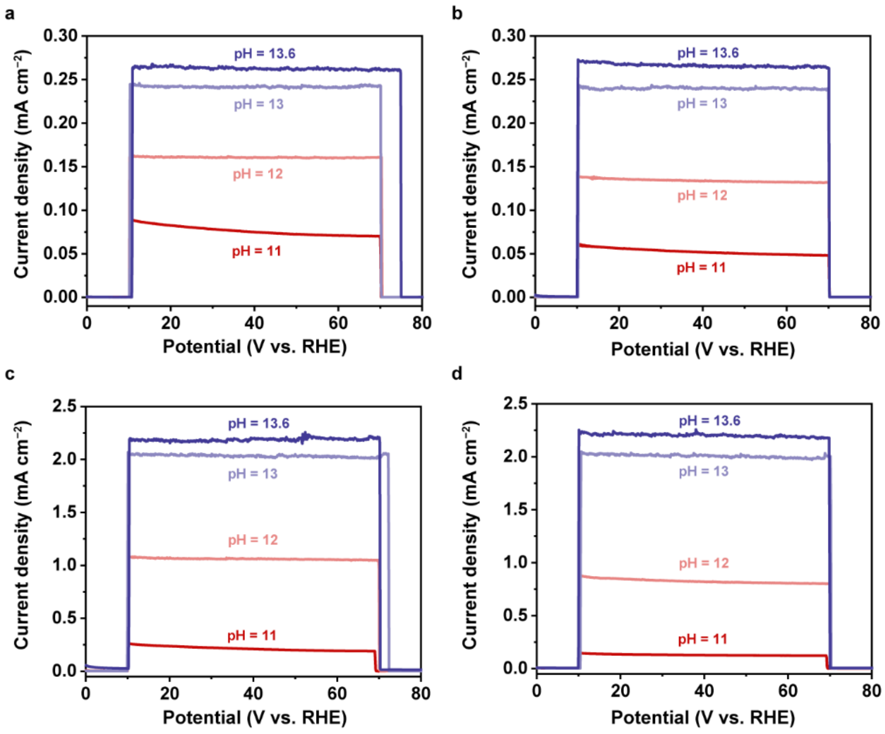

Supplementary Fig. 11. Steady state photocurrent measurement in D_2O.

Stability test of *β*-Fe_2O_3 in **a**, H_2O and **b**, D_2O and Sn/*β*-Fe_2O_3 in **c**, H_2O and **d**, D_2O at different pH values.

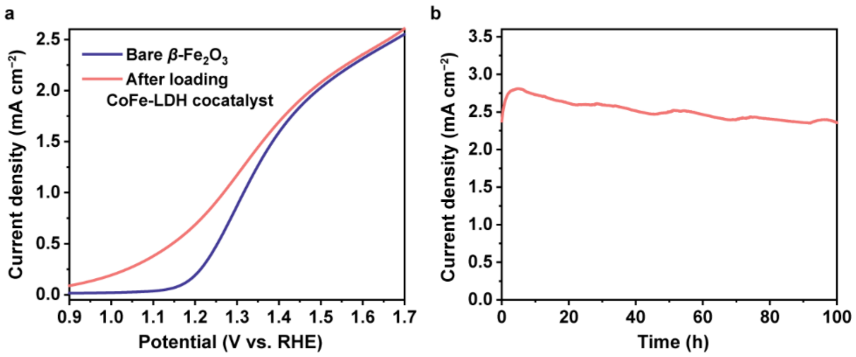

Supplementary Fig. 12. Photoelectrochemical performance of Sn/*β*-Fe_2O_3 before and after loading of the CoFe-LDH cocatalyst.

a, Photocurrent density of bare Sn/*β*-Fe_2O_3 and CoFe-LDH @ Sn/*β*-Fe_2O_3. **b**, Stability curve of CoFe-LDH @ Sn/*β*-Fe_2O_3 (1 cm^2) in simulated seawater for 100 h.

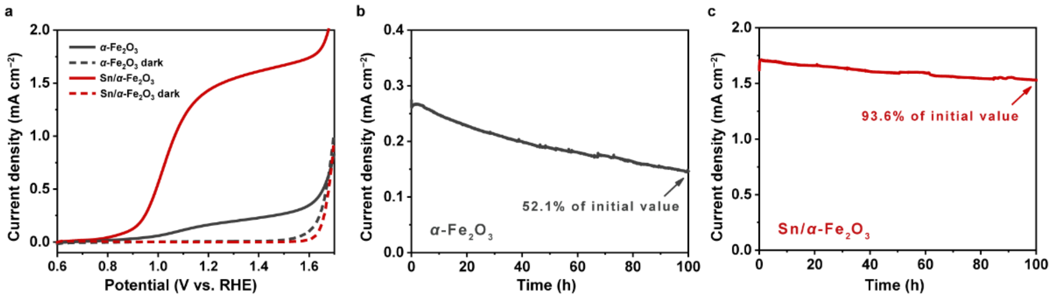

Supplementary Fig. 13. Photoelectrochemical performance of *α*-Fe_2O_3 and Sn/*α*-Fe_2O_3.

a, Photoelectrochemical performances of pure and 2% Sn/*α*-Fe_2O_3 prepared by drop-coating. Stability curve of **b**, *α*-Fe_2O_3 and **c**, 2% Sn/*α*-Fe_2O_3 in simulated seawater for 100 h.

Supplementary Table 1. Comparison of photoelectrochemical OER (simulated) seawater splitting with different photoanodes.

**Photoanodes**	**Light intensity**	**Bias potential**	**Current density**	**Stability**	**Ref.**
Sn/*β*-Fe_2O_3	AM 1.5G	1.6 V_{RHE}	2.21 mA cm^{-2}	3000 h	this work
RhO_2/Mo-$BiVO_4$	Full-arc xenon lamp (λ > 300 nm) with higher light intensity	1.0 $V_{Ag/AgCl}$	18 mA cm^{-2}	270 min	14
TiO_2@*g*-C_3N_4@CoPi	AM 1.5G	1.23 V_{RHE}	1.64 mA cm^{-2}	10 h	38
WO_3/*g*-C_3N_4	AM 1.5G	1.23 V_{RHE}	0.73 mA cm^{-2}	1 h	39
Fe_2O_3/WO_3	AM 1.5G	1.23 V_{RHE}	1 mA cm^{-2}	5 h	8
In_2S_3/ANP/RND	AM 1.5G	1.23 V_{RHE}	1.53 mA cm^{-2}	2 h	9
In_2S_3/In_2O_3	AM 1.5G	0.981 V_{RHE}	~0.2 mA cm^{-2}	1000 s	40
Mg doped ZnO	AM 1.5G	0.5 $V_{Ag/AgCl}$	~1μA cm^{-2}	5 h	41
MoB/$BiVO_4$	AM 1.5G	1.23 V_{RHE}	4.30 mA cm^{-2}	70 h	42
$NiMoO_x$/$BiVO_4$	AM 1.5G	1.23 V_{RHE}	3.0 mA cm^{-2}	190 h	43
Bi_2S_3/NiS/NiFeO/TiO_2	300 W Xe lamp	1.23 V cell voltage	10 mA cm^{-2}	4 h	44
$Bi_{0.6}Fe_{0.4}VO_4$@CNTs	AM 1.5G	1.5 $V_{Ag/AgCl}$	~0.1 mA cm^{-2}	1 h	45

Supplementary Table 2. ICP measurement of Fe ions in the electrolyte after the reaction.

Samples	Fe ion concentration in electrolyte (mg/L)
β-Fe_2O_3	0.071
Sn/β-Fe_2O_3	0.036

We take the alkaline simulated seawater electrolyte after 100 h of reaction, and measure the content of Fe ion in the solution. Based on this, we can analyse the loss of Fe caused by surface reconstruction in the stability test. Dilution and pH adjustment of the same multiple was conducted before the test.
